# Highly pathogenic avian influenza virus (H5N5) detected in an Atlantic walrus (*Odobenus rosmarus rosmarus*) in the Svalbard Archipelago, Norway, 2023

**DOI:** 10.1080/22221751.2025.2456146

**Published:** 2025-01-27

**Authors:** Alexander Postel, Nele Gremmel, Christian Lydersen, Kit M. Kovacs, Luca A. Schick, Ursula Siebert, Ingebjørg H. Nymo, Paul Becher

**Affiliations:** a Institute of Virology, University of Veterinary Medicine Hannover, Hannover, Germany; b Norwegian Polar Institute, Fram Centre, Tromsø, Norway; c Institute for Terrestrial and Aquatic Wildlife Research, University of Veterinary Medicine Hannover, Buesum, Germany; d Marine Mammal Research, Department of Ecoscience, Aarhus University, Roskilde, Denmark; e Food Safety and Animal Health Research, Norwegian Veterinary Institute, Tromsø, Norway; f Department of Arctic and Marine Biology, UiT - The Arctic University of Norway, Tromsø, Norway

**Keywords:** Highly pathogenic avian influenza, virus, infection, walrus, Arctic

## Abstract

We present the first documented case of highly pathogenic avian influenza virus (HPAIV) subtype H5N5 in an Atlantic walrus (*Odobenus rosmarus rosmarus*). The animal was found dead in Svalbard, Norway, in 2023. Sequence analysis revealed the highest genetic similarity with virus isolates from different avian hosts.

Highly pathogenic avian influenza virus (HPAIV) has become endemic in avian populations worldwide. This has resulted in dramatic increases in the numbers of outbreaks in domestic poultry and has caused mass mortality within wild bird populations. Spillover infections are increasingly reported in mammalian species that either prey on diseased birds or scavenge their carcasses. The increasing number of HPAIV infections in pinnipeds may enable the virus to adapt more completely to mammals [1], increasing the risk of zoonotic transmission.

Several dead walruses (approx. 10–20) were reported on islands in the south and along the west coast of the Svalbard Archipelago during summer 2023. Most carcasses disappeared with the next tide, but one animal, found on the remote island of Hopen, was accessible for sampling. Dry swabs were taken from the nose, eyes, and mouth. The swab from the nostril tested positive for influenza A virus using two RT-PCRs targeting the matrix protein and the nucleoprotein-encoding segments [2,3], yielding Cq values of 32 and 34, respectively. Low viral genome loads have previously been observed in the respiratory tissue and secrets of fatally infected seals, despite high concentrations in the brain [2]. This is possibly the case here, with poor sample quality and RNA degradation contributing to low levels of detectable viral RNA.

Although initial attempts of generating longer sequences failed, a one-step RT-PCR using a proofreading polymerase (SuperScript™ IV, Invitrogen) and universal primers [4] followed by molecular cloning, allowed us to obtain the coding sequences for hemagglutinin (HA), neuraminidase (NA) and Polymerase Basic Protein 2 (PB2) from the sample (GISAID database: EPI_ISL_19472336). Sequences of this virus, designated A/walrus/Norway/1/2023, were highly similar to those of several recent isolates of HPAIV subtype H5N5 (H5 clade 2.3.4.4b) from avian hosts in Norway, other northern European countries, Japan, and Canada obtained between 2022 and 2024 (Figure 1). Compared to the most similar avian HPAIV sequences, sequences from the walrus showed differences in three nucleotides (nt) in the H5 and N5 and five nucleotides in the PB2 encoding regions, respectively. Most similar to the walrus H5 sequence is a set of identical sequences from crows obtained in Hokkaido, 2024 (Figure 1, represented by EPI_ISL_19033208). Several N5 sequences with only three different nucleotides include the recent isolates from Hokkaido and sequences from different North European countries (e.g. EPI_ISL_19216501, EPI_ISL_18497662), including also a sequence from a Norwegian eagle (EPI_ISL_18838519). The PB2 sequence obtained from the same eagle is also among most similar PB2 sequences (Figure 1). The first introduction of HPAIV (H5N8) to Norway is thought to have occurred in autumn 2020 [5]. HPAIV was first isolated in the Arctic from a glaucous gull (*Larus hyperboreus*) found in Svalbard in June 2022, it was identified as subtype H5N5 [6]. Comparison of the sequences from this avian Svalbard index case to the walrus sequences showed only very slight differences (H5: 9 nt, N5: 7 nt, PB2: 10 nt, Figure 1).

**Figure 1.**
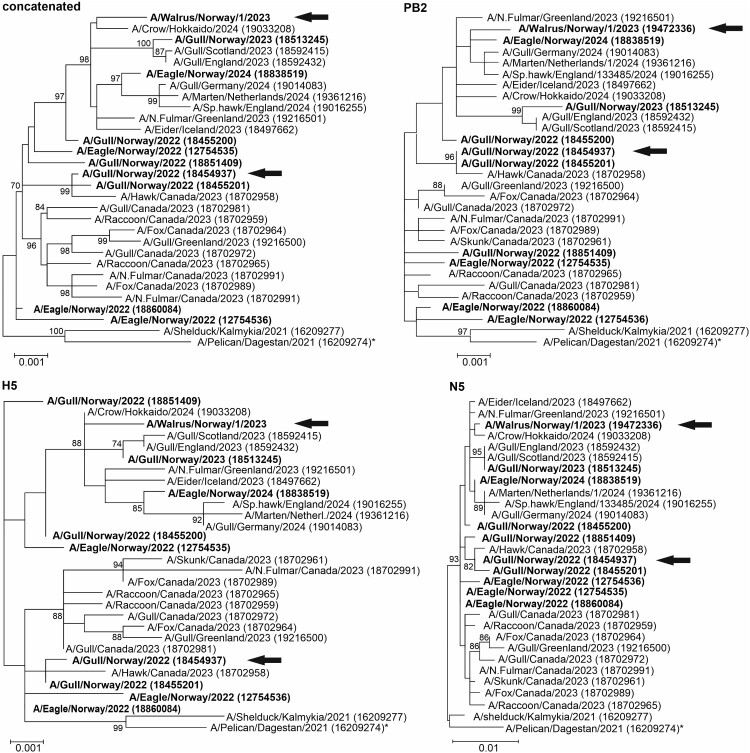
Genetic characterization of the highly pathogenic avian influenza virus (HPAIV) obtained from dead walrus. Maximum-likelihood phylogenetic tree of concatenated and individual Polymerase Basic Protein 2 (PB2), H5, and N5 sequences of selected H5N5 HPAIV. The tree was generated with MegaX using the GTR (G + I) substitution model with 1000 bootstrap iterations (bootstrap values ≥70 are indicated). Sequences originating from Norway are highlighted in bold. Black arrows indicate the sequences of HPAIV from Svalbard. One avian N5 sequence originating from Russia encodes for the complete neuraminidase stalk domain missing a 66 nt deletion present in all other sequences (marked with an asterisk). Numbers in brackets refer to the isolate identifier (EPI-ISL) of the EpiFlu database.

Comparison of A/walrus/Norway/1/2023 with nine complete H5 sequences and one partial H5 sequence (EPI_ISL_12028892, excluded from phylogeny) from Norwegian avian H5N5 infections revealed no amino acid changes in the receptor-binding domain or the polybasic cleavage site (KRRKR/GLF). Variation in the glycosylation site N154–T156 in mature H5 has been observed in avian HPAIV sequences from Norway [7]. The T156A mutation prevents glycosylation and favours binding to mammalian host cells [8]. T156 was maintained in the walrus and three avian sequences, but five avian sequences from Norway contained the T156A mutation. A serine at position 156 was present in two sequences including the avian Svalbard index case. Furthermore, the N5 sequences from the walrus as well as from avian species have a deletion (66 nt) in the region encoding for the stalk domain, which is a virulence determinant in chickens [9]. HPAIV subtype H5N5 containing this deletion likely evolved in the Caspian Sea region and was thereafter spread via birds migrating to Norway and via multiple transatlantic incursions to North America [7,10]. Compared to the Svalbard avian index case, the PB2 segment of the walrus sequence contained only two amino acid substitutions, including the E627K mutation, which is known to enhance infectivity in mammals. Interestingly, all avian HPAIV H5N5 from Norway, as well as the walrus sequence, have a set of six amino acids in PB2 that have been reported to favour infection of mammals [11].

Together with a recently reported fatal infection of a polar bear in Alaska [12], this case in a walrus is one of the first documented HPAIV infections in Arctic mammals. Investigation of 210 Atlantic walruses from Canada (1984–1998) provided no serological evidence of influenza infections [13], although serological investigations have demonstrated the presence of antibodies against influenza viruses belonging to H10, N2, N3 and N7 subtypes in 8 of 38 serum samples from Pacific walruses (*O. r. divergens*) from Alaska (1994–1996) [14]. While HPAIV H5Nx infections have been detected previously in pinnipeds belonging to the families *Phocidae* and *Otariidae*, this is the first detection of HPAIV infection in a walrus (Family *Odobenidae*).

HPAIV infections in pinnipeds and other mammals can result in encephalitis with fatal outcomes. Although our investigations of the available sample material could not confirm a direct link between HPAIV infection and disease in the walrus, it is reasonable to hypothesize that the infection was the cause of death.

Increased exposure to HPAIV, the emergence of new H5Nx variants adapted to mammalian hosts, and the high degree of contact among walruses due to their natural social behaviour could collectively contribute to the rapid spread of this pathogen, possibly leading to devastating consequences for walrus populations.
